# Recent Advances in Applications of Hybrid Graphene Materials for Metals Removal from Wastewater

**DOI:** 10.3390/nano10030595

**Published:** 2020-03-24

**Authors:** Abdulrahman Abu-Nada, Gordon McKay, Ahmed Abdala

**Affiliations:** 1 Division of Sustainable Development, College of Science and Engineering, Hamad Bin Khalifa University, PO Box 34110, Doha, Qatar; aabunada@mail.hbku.edu.qa; 2 Chemical Engineering Program, Texas A&M University at Qatar, POB 23874, Doha, Qatar

**Keywords:** graphene nanocomposites, heavy metals, adsorption, regeneration

## Abstract

The presence of traces of heavy metals in wastewater causes adverse health effects on humans and the ecosystem. Adsorption is a low cost and eco-friendly method for the removal of low concentrations of heavy metals from wastewater streams. Over the past several years, graphene-based materials have been researched as exceptional adsorbents. In this review, the applications of graphene oxide (GO), reduce graphene oxide (rGO), and graphene-based nanocomposites (GNCs) for the removal of various metals are analyzed. Firstly, the common synthesis routes for GO, rGO, and GNCs are discussed. Secondly, the available literature on the adsorption of heavy metals including arsenic, lead, cadmium, nickel, mercury, chromium and copper using graphene-based materials are reviewed and analyzed. The adsorption isotherms, kinetics, capacity, and removal efficiency for each metal on different graphene materials, as well as the effects of the synthesis method and the adsorption process conditions on the recyclability of the graphene materials, are discussed. Finally, future perspectives and trends in the field are also highlighted.

## 1. Introduction

Both natural and anthropogenic sources linked to industrial activities such as electroplating, metal smelting, fertilizer industries, mining operations, pesticides, and paper manufacturing result in wastewater streams contaminated with various heavy metals. Discharging these streams into the environment leads to the absorption and accumulation of heavy metals into living organisms resulting in severe health complications. Therefore, the current stringent regulations, requires the removal of heavy metal from wastewater streams to extremely low levels. The WHO guideline [1] for the quality of drinking water restricts the concentrations of heavy metal to a fraction of mg/L, as shown in Table 1.

**Figure 1:**
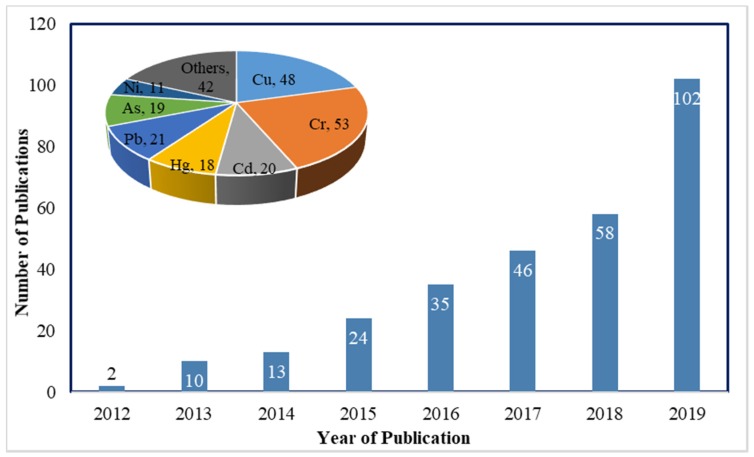
Annual publications returned using “graphene” and “metal removal” as keywords from ScienceDirect. Inset: the number of publications on removal of specific metals.

Several technologies are being used or proposed for removing heavy metals from wastewater streams including chemical precipitation [2], ion exchange [3], membrane filtration [4], flotation [5], electrochemical coagulation treatment [6], and adsorption. Among these methods, adsorption has the advantage of process flexibility, low cost, and the ability to reduce the heavy metal to the required low level, making it the preferred treatment method for heavy metal removal [7]. Both conventional adsorbents such as activated carbon [8,9] and nanoadsorbents including carbon nanotubes [10,11], chitosan [12], and nanocellulose [13] are candidates for heavy metal removal. Recently, graphene- based materials are being explored and proved to be efficient nanoadsorbents for the removal of heavy metals as shown by the number of publications on “graphene metal removal” that increased from 2 publications in 2012 to 102 publications in 2019 with the majority of these publications on removal of copper and chromium as indicated in Figure 1. 

Although graphene can be prepared by bottom up approaches such as CVD, epitaxial growth, templating, and organic synthesis, graphene materials used in adsorption applications are prepared by top-down methods via oxidation of graphite to produced graphene oxide (GO) that can be thermally or chemically reduced to obtain reduced graphene oxide (rGO). Further modification of GO and rGO via the development of nanocomposites with metal oxides and organic molecules enhances their sorption characteristics.

This review focuses on the removal of heavy metals from wastewater using various graphene materials. Although there are already few articles that review the adsorption of various metals on graphene materials, this article is more comprehensive and it also accounts for the large number of very recent articles appeared in 2018 and 2019, which represent about 60% of the total number of publications. Moreover, this review includes a concise section on the synthesis of various graphene materials and composites in order to classify the various graphene materials and establish the correlation between the synthesis method, structure, and the adsorption characteristics of the graphene materials. Moreover, the key adsorption parameters associated with each metal on various graphene-based materials are summarized in tables and the performance of the key graphene materials are highlighted and discussed. 

It is very evident that application of graphene materials as adsorbents for metal removal is a rapidly growing research field as can be seen by the very large increase in number of publications in 2018 and 2019, Figure 1. The adsorptive properties of graphene can be applied in many forms, e.g., sheets, nanotubes, 3D structures, and membranes [14]. These forms have been utilized in water purifications for removal of a variety of contaminants such as heavy metals, different dyes, pharmaceuticals, and organics [15]. It is also worth noting that this review focuses on the adsorptive removal of heavy metal and the use of graphene-based membranes for removal of heavy metals is beyond its scope. 

This review begins with a concise discussion on the synthesis of GO, rGO and their nanocomposites with organic, inorganic, and polymers, which have been used for metal removal. Then an overview of the typical methodology to study adsorption and the models used for analysis of the process thermodynamics and kinetics are provided and a comprehensive analysis of the performance of various graphene-based materials for the adsorption of various metals is carried. Finally, an outline of the major challenges for the commercialization of graphene-based adsorbents for heavy metals and highlights the future research directions are presented. 

## 2. Graphene-Based Materials for Removal of Heavy Metals

The graphene materials used for the removal of heavy metals are classified into two broad categories that include:

1. GO and rGO and their foam/aerogel structures and
2. Hybrid of GO or rGO with metallic, organic, inorganic, and polymeric materials

While there are few studies on the use GO and rGO in powder, aerogel, and 3D foam structure, most of the reports are on using hybrids of GO and rGO with organic, inorganic, and/or polymers. In this section, we summarize the synthesis methods for GO, rGO and hybrid graphene materials.

### 2.1. Graphene Oxide and Reduced Graphene Oxide 

**Figure 2:**
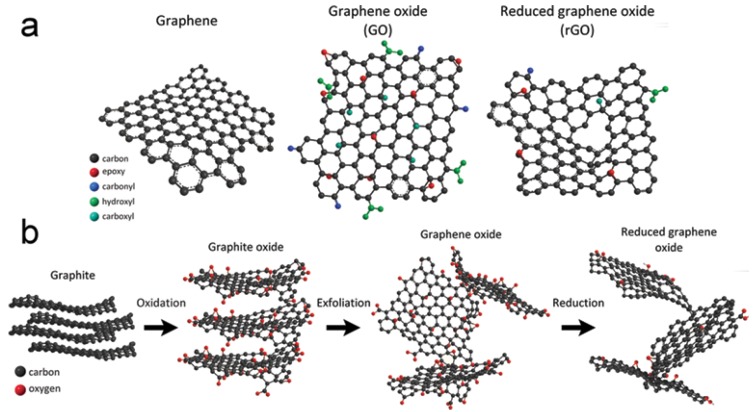
(**a**) Chemical structure of graphene (G), graphene oxide (GO), and reduced graphene oxide (rGO) and (**b**) route of graphite to reduce graphene oxide.(Reproduced or adapted from ref. [20], with permission from Intech, 2016).

Although graphene can be obtained directly from graphite using mechanical cleavage or solution assisted exfoliation, the dominant type of graphene used in adsorption application is prepared via the graphite oxide route not only because its potential for large scale production, but also because it produces a functional form of graphene that is attractive for adsorption applications. In this route, GO is produced by exfoliation of graphite powder, which is produced through solution oxidation/intercalation of graphite using acids and oxidants. The first report on the synthesis of graphite dates back to 1958 by Brodie using nitric acid and potassium chlorate [16]. Currently, the most widely used methods in the preparation of graphite powder are the Hummer’s method [17], modified Hummer’s method [18], and Tour’s method [19]. GO maintains the layered structure of graphite but with expanded interlayer separation due to the presence of polar oxygen groups such as hydroxyl, epoxy, carbonyl, and carboxylic. The introduction of these groups changes the carbon hybridization from sp^2^ in graphite to a mixture of sp^2^ and sp^3^ in GO. The oxygen content of graphite oxide is defined by the C/O ratio which ranges between 1:1 in highly oxidized GO to 4:1 in less oxidized GO depending on the oxidation method and conditions. The presence of these oxygen groups makes graphite oxide very hydrophilic such that it readily exfoliates to GO in aqueous solution. rGO is prepared by thermal, hydrothermal, or chemical reduction of graphite oxide or GO. The reduction of GO to rGO reduced the oxygen content to about C/O ratio of 10. The structure of GO and rGO as well as the schematic for their preparation is shown in Figure 2 [20].

**Figure 3:**
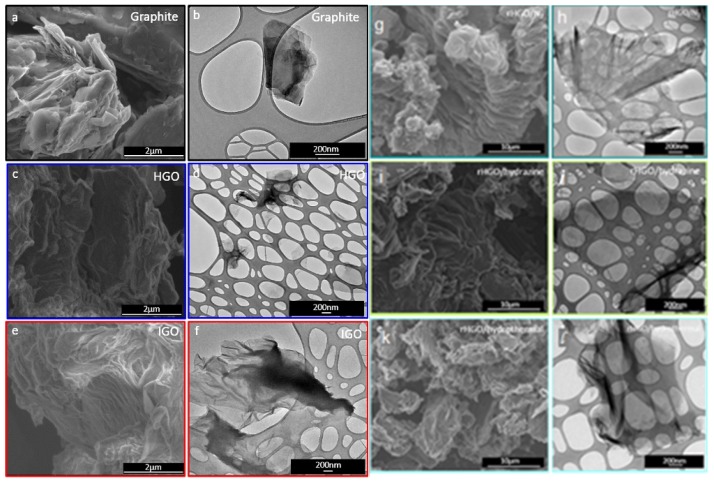
(**a**) SEM and (**b**) TEM of graphite, (**c**) SEM and (**d**) TEM of GO produced by Hummer’s Method, (**e**) SEM and (**f**) TEM of GO produced by improved Hummer’s method, (**g**) SEM and (**h**) TEM of rGO produced by thermal reduction in N_2_, (**i**) SEM and (**j**) TEM of rGO produced by chemical reduction using hydrazine, and (**k**) SEM and (**l**) TEM of rGO produced by solvothermal method. (Reproduced or adapted from ref. [21], with permission from Elsevier, 2020.)

Alazmi et al. [21] investigated the changes in the morphology of GO based on the oxidation method and the structure morphologies of rGO prepared by thermal, chemical, and hydrothermal reduction are probed by SEM and TEM analysis as shown in Figure 3. 

In addition to microscopic characterization of GO and rGO, characterization using spectroscopic techniques such as XPS and FTIR and diffraction techniques such as XRD and SAD are commonly used to determine the structure of GO and rGO and the presence of oxygen-containing functional compounds. Other characterization techniques have been performed. The synthesis of the adsorbents was been carried out with a variety of methods and with great rigor and control. Heat treatment at different temperature ranges, vacuum promoted exfoliation, physical incorporation of additives, and surface modifications using oxidative agents are a few techniques used in the synthesis processes [14]. For more details on the synthesis and characterization of GO and rGO, we refer the reader to the excellent and concise review article by Park and Ruoff [22]. 

### 2.2. Graphene Composites for Adsorption Applications 

To improve the adsorption characteristics of GO and rGO and to enable their effective application in selective adsorption of heavy metals, a few strategies are applied. These strategies include the formation of hybrid structures of GO or rGO with oxides of transition metals such as iron, Zn, Cu, Ni, and Co, organic materials such as cellulose and chitosan, inorganic materials including metal organic framework (MOF), zeolites, and silica nanostructure, polymers and mixtures of two or more of these groups. Among these strategies, the development of magnetic nanocomposites of GO and rGO with Fe_3_O_4_ are very commonly applied not only to enhance the adsorption characteristics, but also to allow the retrieval of the adsorbent for regeneration at the end of the batch adsorption using magnetic recovery. Nevertheless, many studies have further modified the magnetic GO or rGO with other metallic, organic or inorganic materials. A concise review of the synthesis of these different hybrid graphene materials applied for the adsorption of heavy metals is provided.

#### 2.2.1. Magnetic Nanocomposites and Nanocomposites with Other Metals

**Figure 4:**
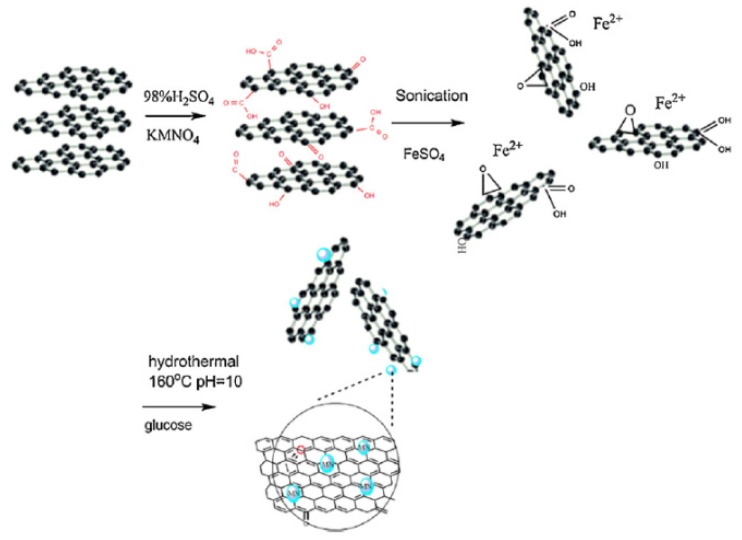
Synthesis of magnetic GO nanocomposite via precipitation method. (Reproduced or adapted from ref. [28], with permission from Elsevier, 2020).

One of the significant challenges faced when using nanosorbents is their separation from the treated solution. Centrifugation, sedimentation, filtration, and other traditional separation methods deem to be ineffective and time consuming. On the other hand, magnetic GO (mGO) is synthesized via impregnation or co-precipitation methods [23,24,25,26,27] and can be easily separated by the aid of magnetic materials like Fe_3_O_4_ or Fe_3_S_4._ mGO is synthesized via impregnation or co-precipitation methods [23,24,25,26,27]. In the co-precipitation method, mGO is synthesized via the hydrothermal process by an in-situ reduction/decomposition of a metal precursor such as Fe_3_O_4,_ FeSO_4_, Fe(NO)_3_, or iron acetate on the surface of dispersed GO under sonication, see Figure 4. This step is frequently followed by an annealing step to enhance the crystal structure of the magnetic nanoparticle and induce complete reduction of GO to rGO. In addition, mGO could also enhance the adsorption of heavy metal ions but the calcination step may have a negative effect on the sorption properties as reducing GO tend to decrease the content of the oxygen functional groups, which in turn decreases the number of binding sites on the surface of the mGO. 

**Figure 5:**
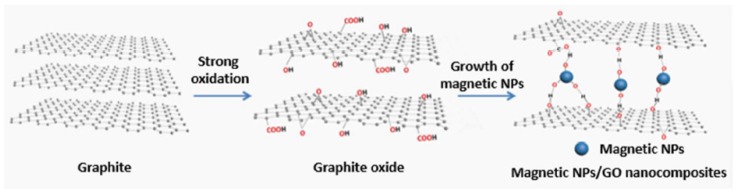
Hydrothermal synthesis of magnetic graphene oxide (mGO) via impregnation of Fe_3_O_4_ particles on the surface of GO flakes (Reproduced or adapted ref. [27], with permission from Royal Society of Chemistry).

In the impregnation method, a GO suspension is mixed with pre-synthesized magnetic nanoparticles and sonicated at room temperature until a homogenous suspension is achieved. The mGO is then collected by centrifugation followed by freeze-drying, Figure 5.

Zhu et al. used ZnSO_4_·7H_2_O to create GO/Brianyoungite GO/BY) composite with hollow spherical and flake-like morphologies. Wen et al. [29] incubated GO into a simulated body fluid for 7 days to ensure mineral growth, which resulted in GO–hydroxyapatite (GO-MWCNT-PDA) 3D flower-like composite. Khatamian et al. [23] fabricated Fe_3_O_4_/GO/Cu-ZEA via solid state dispersion of Cu-ZEA and Fe_3_O_4_ into GO suspension followed by sonication. Lingamdinne et al. [30] mixed Hydrazine hydrate, iron and nickel with GO and heated to 120 °C to make rGO-Nickel Ferrite nanocomposite (rGO-NiFerrite NC). 

#### 2.2.2. Hybrids with Chitosan and Cellulose

**Figure 6:**
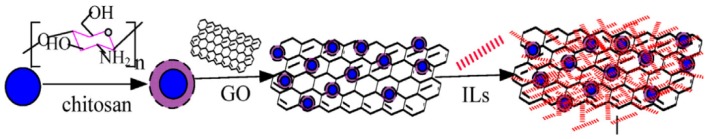
Synthesis route for preparation of GO–chitosan and GO–Chitosan–Ionic Liquid nanocomposites (Reproduced or adapted from ref. [33], with permission from Elsevier, 2020).

Graphene-based hybrids have been widely used in the removal of heavy metals. Some of the materials used to make these composites include chitosan and cellulose. Cellulose is combined with GO to form Ethylenediaminetetraacetic acid (EDTA)-functionalized magnetic chitosan (CS) graphene oxide (GO) nanocomposites (EDTA-MCS/GO) through a reduction precipitation method [31]. This composite is characterized by FT-IR, XRD, SEM, MPMS, zeta-potential and BET analyses and is applicable for the removal of Pb^2+^, Cu^2+^, and As^3+^. Shahzad et al. synthesized EDTA-MCS/GO via 2-step process in which the first step yielded (MCS/GO), which was dried and mixed with Na_2_EDTA to produce the composite [31]. Yang et al. [32] cross-linked GO and rGO with chitosan (CS) to create CS/GO and CS/RGO nanohybrids functionalized with amine and hydroxyl groups. Sun et al. prepared GO-chitosan composite decorated with ionic liquids as shown in Figure 6 [33]. 

#### 2.2.3. Graphene Hybrids with Polymers

**Figure 7:**
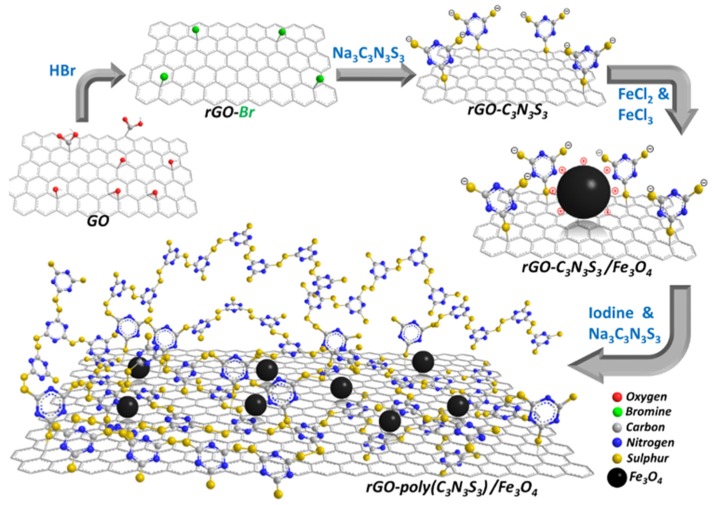
Schematic showing pathway for hybridization of GO with different compounds (Reproduced or adapted from ref. [42], with permission from Elsevier, 2020).

Zhu et al. fabricated a magnetic composite by polymerizing Fe_3_O_4_ and adding it to graphene to develop polypyrrole–graphene (PPy–GO) nanocomposites [34]. Yang et al. polymerized Lignosulfonate and Polyaniline with GO via ultrasonication and vigorous mixing in HCL to produce (LS-GO-PANI) [35]. Tadjarodi et al. added 2-pyridinecarboxaldehyde thiosemicarbazone to GO to create (GO/2-PTSC) assisted with ultrasonic waves [36]. Sulfuric acid doped poly-diaminopyridine/graphene (G-PDAP) was synthesized by Dinda and Saha via the mutual oxidation-reduction technique by adding 2,6-Diamino pyridine to a GO suspension [37]. Najafabadi et al. used glyoxal as a crosslinking agent to develop chitosan/graphene oxide using electrospinning [38]. Li et al. cross-linked DTPA and mGO using diethylenetriamine to produce (DTPA/MGO) [39]. Zare-Dorabei et al. modified graphene oxide with 2,20-dipyridylamine to make (GO-DPA) for the simultaneous adsorption of different metal ions [40]. Nano sized hydrated manganese oxide was grown by Wan et al. [41] on GO to fabricate (HMO@GO), a nanocomposite with a high selectivity for lead. Fu et al. prepared magnetic Fe_3_O_4_-encapsulated poly(C_3_N_3_S_3_) polymer/rGO nanocomposite following the method shown in Figure 7 [42].These are only some of the hybridizations mentioned in this review. Other composites will be discussed throughout the manuscript. 

## 3. Adsorption Measurements and Data Analysis

Water solution containing various concentrations of heavy metal ions are used to carry out the adsorption studies typically at pH 5.5 and room temperature. After equilibrium is achieved, the following parameters are measured or calculated: initial concentration (*C_o_*, mg/L), equilibrium concentration (*C_e_*, mg/L), maximum adsorption capacity (*Q_e_*, mg/g), adsorption percentage (%), the kinetics are studies by measuring the adsorption capacity at a given time t (*Q_t_*, mg/g), solution volume (V, L), weight of GO (m, g), and the final concentration of metal ions (*C_f_*, mg/L). The equations are based on the mass balance of the reaction, as follows:

(1) $$Q_{e}=\frac{\left(C_{o}-C_{e}\right)V}{m}$$

(2) $$Q_{t}=\frac{\left(C_{o}-C_{t}\right)V}{m}$$

(3) $$Removal\;\%=\frac{\left(C_{o}-C_{f}\right)}{C_{o}}*100$$

### 3.1. Adsorption Mechanism

There are two main contributors to the adsorption process. These are electrostatic forces between the negatively charged surface of GO and the positively charged heavy metal ions which leads to ion exchange. Therefore, the adsorption affinity of ions onto GO and rGO surfaces strongly depends on the electronegativity, first stability constant, and standard reduction potential. An example of the order in which metal ions are adsorbed on GO and rGO is: Pb (II) > Cu (II) >> Cd (II) > Zn (II). 

The adsorption affinity is not dependent on the ionic radius of the heavy metals but more on their relative positions in the electro chemical series. However, the capacity can increase or decrease with temperature depending on heat of adsorption, i.e., exothermicity or endothermicity of the process [43]. 

### 3.2. Adsorption Kinetics and Isotherms

Studying the kinetics of the adsorption process is crucial not only to identify the adsorption mechanism but also to ensure the proper design of the adsorption process in terms of the required residence time. In general, experiments are designed to collect data on the change of the adsorbate concentration (*C_t_*) and adsorption capacity (*Q_t_*) versus adsorption time (*t*) and various kinetic models are used to regress these data to find the kinetic model that best describes the collected data. For the adsorption of heavy metal ions on GO-based substances, two kinetic models are commonly applied, pseudo-1st-order kinetic model and pseudo-2nd-order kinetic model.

Adsorption isotherms are important as they express the dependence of adsorption capacities on the equilibrium concentration of adsorbent and adsorbate in the solution and provide information about the mechanism and the interaction between adsorbates and adsorbents. The isotherm models which are commonly used to describe the adsorption capacities and characteristics of heavy metals on different graphene materials are Freundlich, Langmuir, Redlich–Peterson, Sips, and Temkin models. 

### 3.3. Effects of Adsorption Conditions

The main factors affecting the adsorption process are temperature, pH, contact time, adsorbent dosage, and the metal ions initial concentration. Increasing the temperature increases the adsorption rate by diffusion but usually reduces the adsorption capacity. Increasing the adsorbent dosage leads to an increase in the adsorption rate and the percentage of metal ion removal but decreases the specific adsorption capacity. Moreover, an increase in the solid content may lead to “folding” of the GO thus denying access to active binding sites. The initial rate is always high and gradually decreases. Longer contact time ensures that equilibrium is reached and the maximum adsorption capacity at that specific conditions is obtained. The pH of the solution is very important as it can change the charge on the GO surface and the ion species in the solution. At relatively high pH (2 < pH < 10), more functional groups are available and electrostatic interactions between the heavy metal ions and GO are stronger, but the optimum pH for maximum ion uptake may vary depending on the type of the heavy metal ion. 

## 4. Removal of Heavy Metals Using Graphene Materials

### 4.1. Arsenic Removal

Table 2 shows the properties of various graphene-based adsorbents used for removal of arsenate, their adsorption properties and the applicable isotherm and kinetic models. The most utilized graphene-based material is their nanocomposites with metal oxides, inorganic, and organics molecules, with scattered performances with a slight skew towards metal oxide composites. These composites are mostly synthesized via hydrothermal treatment, solid state dispersion, aqueous thermal treatment, with the best performing composites are synthesized hydrothermally. 

**Figure 8:**
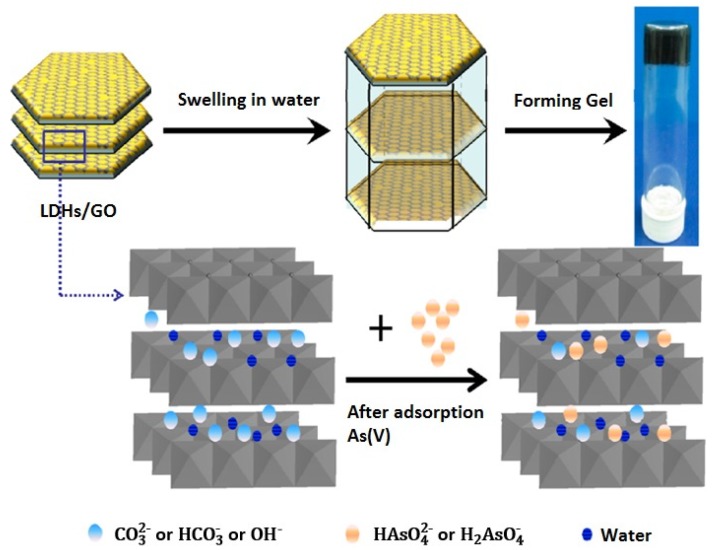
Mechanism for the adsorption mechanism of Arsenic on GO-LDH (Reproduced or adapted from ref. [52], with permission from American Chemical Society, 2020).

GO modified with metal oxides and organic molecules are among the highest capacities, while inorganic composites (Mg−Al layered double hydroxides GO nanocomposite) when dispersed in water, it swells and forms viscous gel as shown in Figure 8 [52]. The high capacities in Table 2 demonstrate the excellent performance of the graphene-based materials very good capacities compared to the maximum reported capacity of other adsorbents including nanochitosan (96.5 mg/g) [54,55], amino-functionalized cellulose (75.1 mg/g) [56], and rice husk (27.8 mg/g) [57].

The kinetic of the arsenate adsorption on the various graphene materials provided in Table 2 follows with no exception pseudo second order kinetics, but the equilibrium adsorption results are best described by Langmuir isotherm with few exceptions where Sips and Freundlich models provided better fitting of the adsorption results for the cases of very low adsorbate concentration, e.g., C_0_~1 mg/L. 

An important and key aspect for the commercial application of the adsorption processes that are based on costly adsorbent, which is often neglected in research studies, is the regeneration and re-usability of the adsorbent. Few studies have investigated the regeneration and reuse of the graphene adsorbents using either a strong alkali (NaOH) or strong acid (HCl) in the desorption step. For As (III), M-nOG, EDTA-MCS/GO, and GO-f were found to be reusable. M-nOG was observed to retain 94.5% of the first cycle capacity after 5 cycles [51]. Moreover, EDTA-MCS/GO depicted high levels of magnetic sensitivity in the presence of an external magnetic field after 4 cycles which indicated good reusability status [46]. On the other hand, GO-f’s removal efficiency decreased by 11% after 3 cycles [44]. For As (V), M-nOG was found to be reusable and retained more than 92% of the first cycle capacity after 5 cycles [51]. 

### 4.2. Lead Removal

**Figure 9:**
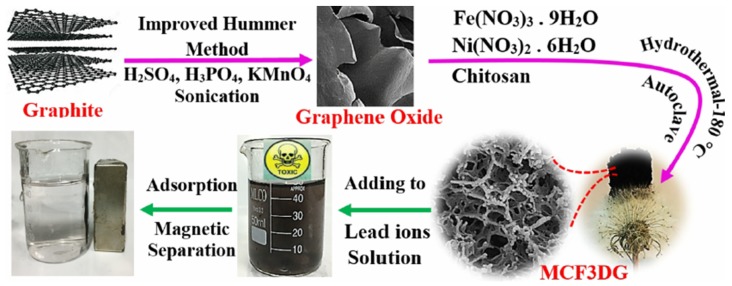
Synthesis of magnetic chitosan-functionalized 3D graphene nanocomposite MCF3DG (Reproduced or adapted from ref. [62], with permission from American Chemical Society, 2020).

Because of the toxicity and abundance of lead in wastewater streams, there are large number of studies focused on the adsorption of Pb (II) on GO with several of studies investigating the regeneration of the graphene adsorbents. The properties of the graphene adsorbents and their lead (II) adsorption properties are summarized in Table 3. Graphene composites with inorganic materials represent the majority of the adsorbents used for Lead removal. Hybrids with Fe_3_O_4_ [41,48,58,59,60,61] was very popular choice due to its magnetic properties that allow separation of the adsorbent as shown in Figure 9 [62]. 

The trends for the best synthesis method to yield a high capacity adsorbent were very hard to distinguish. The best capacities were achieved by modifying graphene with polymers and inorganics [63,64]. The majority of the composites were synthesized hydrothermally or with dispersion reaction. Solvothermal synthesis was used to composite inorganics with graphene [63]. Adsorbents based on combination of metal oxide and inorganics with graphene were synthesized via hydrothermal routes [30,62,65,66]. No strong correlation between the adsorbent surface area and the adsorption capacity of these composites can be made [53,59,67].

The most economic synthesis methods are those using fewer chemicals and production steps. One-pot hydrothermal, dispersion and co-precipitation with few components would serve as a better synthesis option especially when coupled with good performance. For instance, 3D G/chitosan/nickel ferrite NC was synthesized hydrothermally in a few steps utilizing sustainable components like chitosan and is one of the best performing graphene-based adsorbents in the removal of lead from aqueous solutions with a maximum capacity of 957.3 mg/g [62]. 

The potential for the regeneration of the lead loaded adsorbents has also attracted much attention and several studies have investigated the reusability of the sorbents after regeneration. These include: CoFe_2_O_4_-G, NiFe_2_O_4_-G, Fe_3_O_4_-GS, EDTA-mGO, EDTA-MCS/GO, DTPA/MGO, GOMCS-IL, and IT-PRGO, Mg−Al LDH / pRGO [67,68,69,70,71,72,73,74,75]. Most of these nanocomposites were reusable with Fe_3_O_4_-GS and EDTA-mGO maintained more than 80% of their original adsorption capacity [76,77]. The best regeneration properties reported belong to mGO-PAMAM, which retained the adsorption capacity after 5 cycles [70]. EDTA-MCS/GO depicted very high magnetic sensitivity in the presence of magnetic field after 4 cycles which indicates good reusability status [46]. The adsorption efficiency of DTPA/MGO was reduced by only 15.5% after 6 cycles signaling good reusability [39]. Similarly, GOMCS-IL suffered a slight loss in adsorption capacity after 4 cycles of adsorption-desorption, indicating good recyclability of GOMCS-IL [33]. IT-PRGO was also reusable and very efficient in adsorbing Pb (II) [47]. Mg−Al LDH/ pRGO was effectively regenerated using HCl as an eluting agent and was reusable without loss in removal efficiency [26]. NiFe_2_O_4_-G and CoFe_2_O_4_-G had 98% and 100% desorbing capacity and the adsorption efficiency was almost retained during 3 repeated cycles [64,66]. Basic magnetized graphene oxides are also used to remove lead ions and showed maximum adsorption capacity of 326.7 mg/g [70]. It worth noting that the capacities for these graphene materials for lead vary not only with the material type and composition but also the conditions like pH, surface area and lead concentrations as shown in Table 2.

**Table 3:** Adsorption properties of multiple GO-based adsorbents used in Pb (II) removal.

Modification	Adsorbent	Surface Area (m^2^/g)	G/Pb Ratio (g/g)	pH	Max. Pb Conc. (mg/L)	Capacity (mg/g)	Max. Removal (%)	Ads. Isotherm	Ref.
NO	GO	-	1.6	-	60	120	98	-	[78]
	Surface-Modified G	101	4	5	1000	196	-	L	[79]
Inorganic	G-Fe	201.3	1	6	100	645	-	L	[80]
	TI/GO@Fe_3_O_4_	0.2	0.4	5	500	461	-	L	[74]
	SGO	-	-	-	-	415	-	L	[81]
	ZIF-8@GO	946.5	-	5	30	356	70	L	[82]
	GO-SO_x_R@TiO_2_	208	-	-	-	312	-	L	[83]
	Fe_3_S_4_/rGO	80.9	7.5	6	500	285.7	80	L	[84]
	GO-SO_x_R	102	-	-	-	285	-	R-L	[83]
	GO-SO_x_R@SiO_2_	92	-	-	-	172	-	L	[83]
	PG-C	154.5	-	7	1	131.4	99.8	L	[85]
	rGO-NiFerrite NC	167.26	40	5	10	121.9	99	L	[30]
	Mg−Al LDH/ pRGO	79.4	0.2	4.5	100	116.2	100	L	[26]
	pRGO	50.2	-	4.5	-	65.0	100	L	[26]
	CNF-C	45.7	-	7	1.5	42.9	48	L	[85]
	GO-OMS-20	872.9	1	-	100	39.5	78.7	L	[53]
	Cu(tpa).GO	-	0.29	7	35.1	37	-	L	[86]
	CdS-G / ZnS-G	-	666.6	5.9	1.5	3.1	99	L	[87]
Inorganic + Metal Oxide	GONF	136	0.75	5.5	1000	10.8	93	L	[88]
	MCF3DG	-	0.064	8.5	26	957.3	100	L	[62]
	GO-W-MC	-	0.03	7	300	253.2	89	L	[65]
	MGL	74.9	1.81	5	550	63.3	97.5	L	[66]
	rGO-NiFerrite NC	167.3	40	5	10	25.8	>99	L	[30]
Metal Oxide	HMO@GO	383.9	-	5	-	553.6	100	F	[41]
	MNGH	156	0.08	5	250	356.4	-	L	[58]
	GNPs/Fe-Mg-Cu	104.9	120	7	-	251.3	99	F	[89]
	GOMO	623	5	6.5	80	190	-	L	[59]
	CoFe_2_O_4_-G	126.36	8.3	5	30	142.8	100	L	[60]
	NiFe_2_O_4_-G	57.11	8.3	5	30	111.1	100	L	[60]
	G−ZnO	-	100	6	10	23.4	92	L	[90]
Organic	L-Glu/GO	-	0.6	5	2000	513.4	-	L	[63]
	Chitosan/GO	-	0.5	3	1000	461.3	-	R–P	[38]
	MWCNT-PDA/GO	356.1	0.167	6	400	350.9	-	L	[91]
	PAamidoamine-g mGO	-	-	6	-	326.7	92.6	L	[70]
	DTC-GO	-	5	5.3	50	132.0	-	L	[92]
	IT-PRGO	-	0.08	5	400	101.5	98	L	[47]
	GOMCS-IL	357	5	5	200	85	-	L	[33]
	GO-TETA-DAC	762	-	5	100	80.9	77	L	[67]
	Pb-MCGO	392.5	-	5	-	79	90	L	[93]
	MCGO	382.5	-	5	-	77	-	L	[94]
Organic + Metal Oxide	EDTA-mGO	49.9	0.5	4.1	100	268.4	95.5	F/T	[76]
	GO/Fe_3_O_4_-g-G	-	-	5	-	181.4	-	L	[95]
	rGO-PDTC/Fe_3_O_4_	194.8	0.25	6	100	147.1	-	L	[68]
	RGO/Fe_3_O_4_	58	-	5.5	30	48	96	L	[61]
Polymer	g-C-EN-GO	-	0.08	7	3127	186.5	55.1	L	[96]
	GO+Zn@NH_4_Cl	-	1	-	635	17900	99	F	[97]
	RGO/PAM	-	0.6	6	1500	1000	-	L	[64]
	GOCA	-	-	5	200	602		L	[98]
	EDA-RGO	28	0.5	7	200	413.2	-	L	[99]
	DTPA/MGO	-	0.025	3	400	387.6	-	L	[39]
	MMSP-GO	-	5	9	20	333	-	L	[100]
	PAS-GO	53.7	0.025	4.9	400	312.5	100	L	[101]
	LS-GO-PANI	-	0.4	5	1000	216.4	98.3	L	[35]
	GO Fe_3_O_4_- DETA	-	-	5.5	0.11	172.4	100	L	[102]

L: Langmuir, F: Freundlich, T: Temkin, R–P: Redlich–Peterson.

Despite the large number of adsorption studies for the removal of lead (II) using graphene oxides and derivatives of graphene oxide, almost all the equilibrium adsorption results are best-modeled using the Langmuir isotherm. Most of the kinetic results follows pseudo-second order kinetics with exception of Mg-Al LDH/pRGO and pRGO, which followed Elovich kinetics model [26], HMO@GO that followed the Intraparticle Diffusion kinetics [41], and Chitosan/GO that followed the Double-exponential kinetic model [38]. Moreover, as shown in Table 2 the adsorption capacity of lead (II) on various graphene materials are exceptionally high and ranged between 150 and 1000 mg/g, which are much higher than the capacity on other adsorbents including coconut-based activated carbon (83 mg/g) [103], activated Melocanna baccifera Roxburgh charcoals (bamboo) (77 mg/g) [104], chitosan (115 mg/g) [105], sphagnum peat moss (75 mg/g) [106], e-waste exchange resin and Lewatit (721 and 391 mg/g) [107], and steel-making dust (209 mg/g) [108]. In one report, the measured capacity of Lead on GO+Zn@NH_4_Cl was 17,900 mg/g which is extremely high, although the conditions and results of the study may not be realistic [97]. 

### 4.3. Cadmium Removal

The adsorption capacity, removal efficiency, and the isotherm models for the adsorption of Cadmium (II) on different graphene materials are summarized in Table 4. The reported adsorption capacity ranged between 116 to 386 mg/g for high initial Cadmium (II) concentrations, the adsorption kinetics followed the pseudo-2nd order, and the adsorption thermodynamics is consistent with the Langmuir isotherm. 

The most common routes used for synthesis of graphene composites used for Cadmium removal were the solvothermal, hydrothermal, dispersion, and co-precipitation methods. However, nanoscale zero-valent iron-reduced graphene oxide composite was prepared via plasma-reduction method and graphene oxide embedded calcium alginate was prepared via drop-wise formation method to create bead like structures [109]. The best performing adsorbent reported applied an inorganic modification to graphene oxide and so did the second best [83,108]. Nevertheless, these adsorbents required extensive processes to synthesize, not taking into consideration the steps required for basic GO. This may have a negative impact on the cost and could increase it dramatically especially when taking scale-up into consideration. 

**Figure 10:**
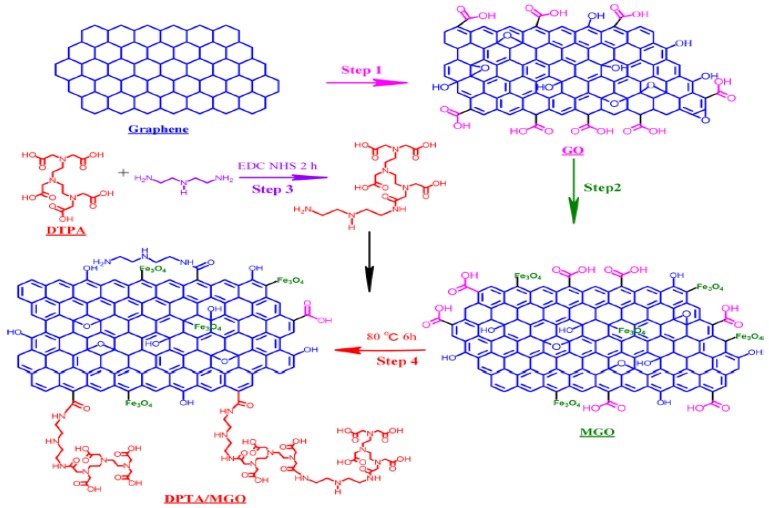
Synthesis Processes and Chemical Structure of DTPA/MGO (Reproduced or adapted from ref. [39], with permission from American Chemical Society, 2020).

Regeneration and reusability of the graphene materials are examined by several groups and mGO- DTPA, G-NiFe_2_O_4_, G-CoFe_2_O_4_, GO-DPA, rGO-NZVI and GO-f are found to be reusable with a slight decrease in capacity with number of cycles. For example, the Cadmium adsorption capacities of DTPA/MGO-based composites synthesized following the route shown in Figure 10 decreased by only 10.3% after 6 cycles [39]. G-NiFe_2_O_4_ and G-CoFe_2_O_4_ retained 98% and 100% of their capacity during 3 repeated cycles [60]. GO-DPA also maintained more than 80% of its removal efficiency after 3 cycles [40]. rGO-NZVI did not show any significant decrease in efficiency after 4 cycles of adsorption and desorption [109]. The recyclability of GO-f after 3 cycles was comparable to those reported in literature with 89% of the maximum capacity achieved [44]. 

Magnetized DTPA-mGO has been synthesized by combining Fe_2_O_3_, GO and DTPA forming a compound with a higher metal binding and electrostatic forces thus improving adsorption capacity [31].

Cadmium is another highly toxic heavy metal, where the kinetics of its adsorption with various conventional adsorbents including Amberlite IR-120 synthetic resin—101 mg/g [110], modified sugarcane bagasse pith—188 mg/g [111], hydroxyapatite—181 mg/g [112], modified magnetic chitosan—202 mg/g [113], magnetic dithiocarbamate—83.21 mg/g [68], and e-waste derived alumino-silicate ion exchange resin—235 mg/g [114] are represented by the pseudo-second order model. In comparison, GO-based adsorbents had better results overall with capacities ranging from 3.6 mg/g to 426 mg/g. In a highlight case, a huge capacity of 8.4 g/g was reached where the adsorption process happened using the in-situ reduction of GO with NH_4_Cl and Zinc [97]. The entire list of GO-based adsorbents followed the pseudo second order kinetic model. 

**Table 4:** Adsorption properties of multiple GO-based adsorbents used in Cd (II) removal.

Modification	Adsorbent	Surface Area (m^2^/g)	G/Cd Ratio (g/g)	pH	Max Cd Conc. (mg/L)	Capacity (mg/g)	Max Removal (%)	Ads. Isotherm	Ref.
None	GO nanosheets	-	5	6	100	167	-	L	[115]
Inorganic	NZVI/rGOs	117.9	-	5	-	425.7	100	L	[109]
	GO-SOxR@TiO_2_	208	-	-	-	384	-	F	[83]
	Surface modified G	101	4	2–8	1000	199.9	-	L	[79]
	Cu(tpa).GO	-	0.3	7	35.9	53	-	L	[86]
	GO-OMS-20	872.9	1	-	100	48	96.9	L	[53]
	nZVI/rGO	-	12	6.5	50	46.5	82.4	L	[116]
	CdS-G/ZnS-G	-	1000	5.9	1	3.6	97	L	[87]
Metal Oxide	rGO/magnetite/Ag NHs	-	1.8	4	112.4	386.8	100	L	[22]
	GO-MO	383.9	0.02	6	500	202	99	F	[117]
	aminoMGO	189.9	-	7	-	184.7	-	L	[118]
	MMSP-GO	-	5	9	20	167	-	L	[100]
	rGO-PDTC/Fe_3_O_4_	194.8	0.3	6	100	116.3	-	L	[68]
	CoFe_2_O_4_-G	126.4	0.8	7	30	105.3	80	L	[60]
	mGO	44.5	7	7	-	90.2	-	L	[69]
	NiFe_2_O_4_-G	57.1	0.5	7	50	74.6	50	L	[60]
	GOFe_3_O_4_-DETA	-	-	5.5	0.12	59.9	99.7	L	[102]
	GO	-	0.01	6	1000	23.9	-	F	[119]
	DTPA/MGO	-	0.03	3	300	286.5	-	L	[39]
Organic	GO-f	-	16.7	8.8	20	285.7	100	L	[44]
	GO-DPA	-	0.4	5	100	257.2	85	L	[40]
	PNGF	-	0.3	-	100	254.9	70	-	[120]
	GOCA	-	-	5	200	181	-	L	[98]

L: Langmuir, F: Freundlich.

### 4.4. Nickel Removal

**Figure 11:**
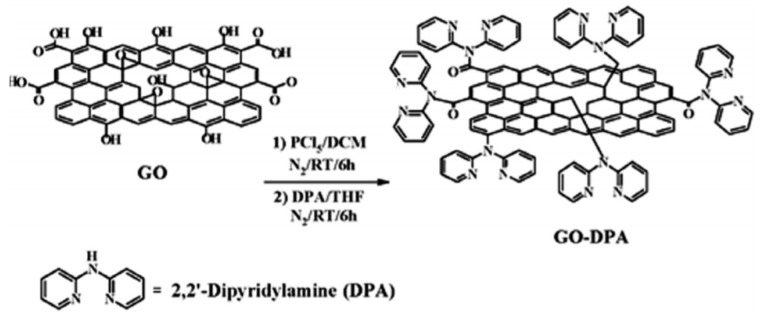
Synthesis Processes and Chemical Structure of GO-DPA (Reproduced or adapted from ref. [40], with permission from Royal Society of Chemistry, 2020).

The adsorption properties of Ni (II) on graphene-based materials are presented in Table 5. Although there are fewer reported studies on nickel than most of the other heavy metals, the trends are very similar to the previous heavy metal studies. All the kinetic studies follow the pseudo-second order out of the five literature studies, four equilibrium models use the Langmuir isotherm, while one model, with a very low initial nickel (II), ion concentration of 5 mg/L, reports a Freundlich isotherm fit. The adsorption of nickel (II) is enhanced at pH 2.0–5.5 owing to the electrostatic force between the negatively charged graphene oxide and positively charged Nickel (II) ions [121]. At pH higher than 7.5, electrostatic repulsion occurs between the negatively charged Ni(II) and GO, which is why adsorption is greatly decreased at pH higher than 7.5 [121]. The highest adsorption capacity is 344 mg/g with GO-SO_x_R@TiO_2_ [71]. The adsorption capacity results for the removal of nickel from water show a dependence on the surface area. There is only a limited number of papers reporting nickel removal by GO derivatives. The regeneration of adsorbent is critical issue, yet only GO-DPA synthesized as described in Figure 11 has been tested for number of adsorption regeneration cycles and > 80% of the adsorption efficiency was retained after 3 cycles proving its potential reusability [40].

The synthesis process of 2, 20-dipyridylamine (DPA) was a simple precipitation in the presence of nitrogen gas. Dry dichloromethane and phosphorous pentachloride were added to GO and mixed to yield a highly sensitive and stable composite for the adsorption of nickel [122]. 

**Table 5:** Adsorption properties of multiple GO-based adsorbents used in Ni (II) removal.

Modification	Adsorbent	Surface Area (m2/g)	G/Ni Ratio (g/g)	pH	Max Ni Conc. (mg/L)	Capacity (mg/g)	Max Removal (%)	Ads. Isotherm	Ref.
I	GO-SO_x_R@TiO_2_	208	-	-	-	344	-	R–P	[91]
O	GO-DPA	-	0.4	5	100	180.9	>85	L	[40]
O	GO+P. Cateniannulatus	185.6	0.833	4	60	104.2	95	L	[121]
MO	Fe_3_O_4_/rGO	109	3.91	5.5	51.1	76.3	100	L	[123]
-	GO	-	-	4	25	35.6	-	L	[124]
MO	Fe_3_O_4_-GS	62.4	2	7	5	22.1	86	F	[77]

I: Inorganic, O: Organic, MO: Metal Oxide L: Langmuir, F: Freundlich, R–P: Redlich–Peterson.

The reported capacities of 76, 104, 181 mg Ni (II)/g GO are linked to the high initial adsorbate concentrations of 51, 60, 100 mg/L, respectively [116]. These values compare favorably with reported values for other adsorbents but are not as high as certain ion exchangers. The nickel adsorption properties on conventional adsorbents include silica-immobilized biomass (4.1 mg/g) [125], histidine modified chitosan beads—56 mg/g [126], protonated rice bran—102 mg/g [127], pine cone biochar—115 mg/g [128].

### 4.5. Mercury Removal

The results of studies on the adsorption characteristics of Hg (II) ions on GO (s) are presented in Table 6. The Table shows that all the kinetic studies were pseudo-2nd order model. Most equilibriums follow the Langmuir isotherm except for five Freundlich systems; however, three of the Freundlich isotherms were for studies performed at very low mercury ion concentrations so the equilibrium saturation level is reached. The other two Freundlich isotherms were applicable for GO-complexes, so possibly more than one adsorption mechanism is involved. The best performing graphene composites for mercury removal were modified with organic compounds. The five highest capacities reported all utilizes organic compounds in preparation of the adsorbents [36,44,47,76,129,130].

All studies were on batch experiments. The most common synthesis methods were hydrothermal, solvothermal, and diffusion methods. Some adsorbents were fabricated through a mechanical route whether ultra-sonication [35], electrospinning [50], or drop-wise formation [98]. There was no evident relation between surface area and maximum capacity. 

High adsorption capacities of mercury in the range of 168–700 mg/g have been obtained with different graphene composites [34,36,47,129,130,131,132,133,134].

**Table 6:** Adsorption properties of multiple GO-based adsorbents used in Hg (II) removal.

Modification	Adsorbent	Surface Area (m^2^/g)	G/Hg Ratio (g/g)	pH	Max Hg Conc. (mg/L)	Capacity (mg/g)	Max Removal (%)	Ads. Isotherm	Ref.
Polymer	Magnetic PPy–GO	1737.6	0.9	7	100	400	99	L	[35]
	GOCA	-	2	5	50	374	97	L	[98]
	Fe_3_O_4_-GS	62.4	2	7	5	23.0	93	F	[77]
Inorganic	Ag/graphene	251	1	5	100	91.7	98.8	F	[129]
	GO-OMS-20	872.9	1	-	100	49	98.5	L	[53]
	GO/Fe-Mn	153.4	12	7	5	32.9	89	S	[130]
Organic + Inorganic	GMA-1	-	-	-	-	719	-	-	[135]
	Cellulose-GNC	14.9	12	6	50	410	77	F	[136]
	GCS	157	-	6	0.89	299.4	80	F	[50]
	SGO/Fe-Mn	-	0.5	6	100	233.4	95.6	BET	[132]
Organic	IT-PRGO	-	0.4	5	900	624	96	L	[47]
	GO/2-PTSC with US	-	0.3	5	90	555	>85	L	[36]
	EDTA-mGO	49.9	1.2	4.1	100	508.4	94.9	F /T	[76]
	Phytic acid induced GO	-	-	7.2	-	361	97	L	[133]
	SeCA-GH	-	0.9	3	100	331	99	L	[137]
	GO-f	-	16.6	8.3	20	227.3	100	L	[44]
	rGO-PDTC/Fe_3_O_4_	194.8	0.3	6	-	158.7	-	L	[68]
	HT-rGO-N	386	0.2	5	11	63.8	98	F	[138]

L: Langmuir, F: Freundlich, T: Temkin, S: Sips, BET: Brunauer–Emmett–Teller.

The Hg (II) capacities on conventional adsorbents are frequently much lower with adsorption capacities ranging from single digit values to over 200 mg/g. Other reported capacities on conventional adsorbents include walnut shell derived activated carbon—151 mg/g [139].

**Figure 12:**
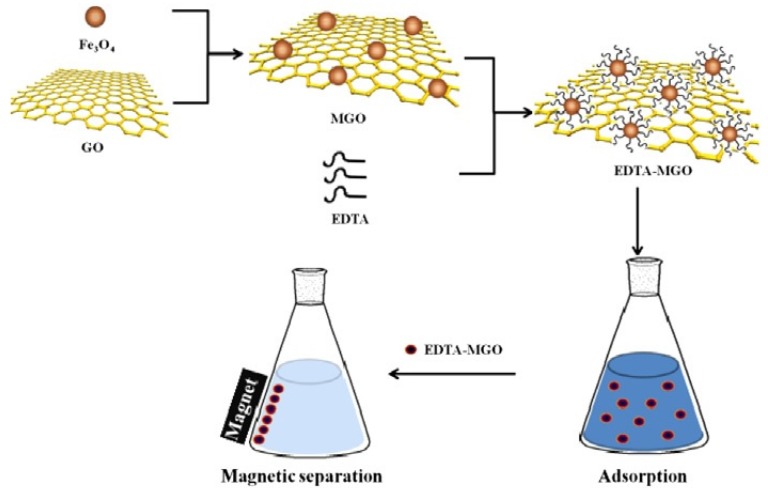
Adsorption and separation process of mercury on EDTA-MGO (Reproduced or adapted from ref. [76], with permission from Elsevier, 2020).

Utilization of the magnetic properties of Fe_3_O_4_ to facilitate the decontamination process by simplifying the separation process described in Figure 12. 

Composite adsorbents have higher adsorption capacities than pure graphene oxide. A novel adsorbent was designed by chemically modifying graphene oxide (GO) with 2-pyridinecarboxaldehyde thiosemicarbazone (2-PTSC) as ligand for removing Hg (II) from aqueous solutions with capacity reaching 555 mg/g. During this experiment the effect of pH, mercury concentrations and time on adsorption capacity were investigated. Moreover, EDTA-mGO, Ag/graphene, GO/2-PTSC, GO/2-PTSC with US, GO-f were identified as reusable adsorbents. Almost half of the studies investigated the regeneration of the adsorbents. Some studies reported excellent regeneration after 6 cycles but had lower maximum capacities in comparison to the rest of the adsorbents [129,137]. Reusability studies on Ag/graphene indicated that the removal efficiency was more than 95% after 6 cycles [129]. Studies on EDTA-mGO showed that removal capacity remained at 85.9% after five cycles showing best recycling properties amongst all [76]. GO/2-PTSC and GO/2-PTSC with US were found to have similar desorption results to all reusable adsorbents in literature with 87% after 3 cycles [36]. Finally, the adsorption efficiency of GO-f was found to only decrease 21% after five cycles [44]. 

### 4.6. Chromium Removal

The adsorption capacities of Cr (III) and Cr (VI) on various GO-based adsorbents are presented in Table 7. The equilibrium adsorption results for most of the graphene adsorbents followed the Langmuir model with a few exceptions that followed Freundlich model and were attributed to a low initial chromium solution concentration or the fact that Fe_3_O_4_ was a component additive and the resulting adsorbent material had more than one active surface adsorption site. On the other hand, the kinetic data are well represented by the pseudo-2nd order model with two exceptions where the double-exponential and intraparticle diffusion models were followed [140].

The double exponential kinetic model assumes the adsorption reaction occurs in two steps comprising a fast stage step 1 and a slow stage step 2, with first order rate constants, *k_A_* and *k_B_*, for each step respectively. This model is represented by Equation (4):

(4) $$q_{t}=q_{e}\;–\;\left(d_{A}/m\right)\;exp\;\left(-k_{A}t\right)\;–\;\left(d_{B}/m\right)\;exp\;\left(-k_{B}t\right)$$

where *q_t_*—amount of chromium adsorbed at time t, *q_e_*—amount of chromium adsorbed at equilibrium e. *d_A_* and *d_B_*—amounts of rapidly and slowly adsorbed fraction of chromium (mg/L), respectively, *m*—mass of adsorbent.

**Figure 13:**
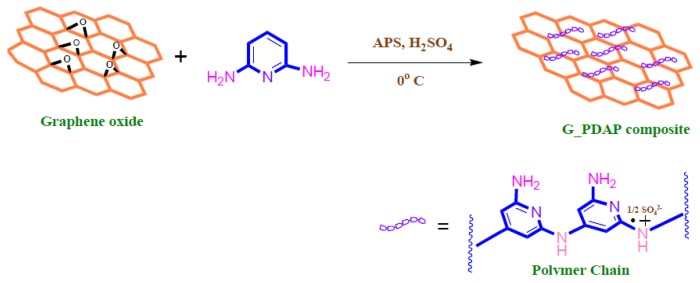
Synthesis process of G-PDAP composite (Reproduced or adapted from ref. [37], with permission from Elsevier, 2020).

G-PDAP is a sulfuric acid doped diaminopyridine polymer prepared in-situ on the surface of GO through mutual oxidation-reduction process as detailed in Figure 13. At the first step, graphene oxide is prepared from graphite through modified Hummer’s method like in most cases and diaminopyridine is then added in the presence of H_2_SO_4_ which introduces more active sites on the surface of the adsorbents [37].

Ultrasonication was a popular synthesis route with organic graphene composites with competitive results [38,80,141]. Nevertheless, the most common synthesis route is hydrothermal. Polymer/graphene composites showed the best performance. Surface areas of the adsorbents were not always reported in the studies, yet when comparing the results, no relation was detected with capacity.

**Table 7:** Adsorption properties of different GO-based adsorbents used in removal of Cr (IV) removal.

Modification	Adsorbent	Surface Area (m^2^/g)	G/Cr Ratio (g/g)	pH	Max Cr Conc. (mg/L)	Capacity (mg/g)	Max Removal (%)	Ads. Isotherm	Ref.
Inorganic	rGO-NiFerrite NC	167.3	40	4	10	126.6	99	L	[142]
	G-Fe-Pb	201.3	0.5	6	22	100	100	L	[80]
	PG-C	154.5	-	6	0.5	68.9	100	L	[85]
	GO-OMS-20	872.9	1	-	100	45.5	96	L	[53]
	GONF	136	0.5	5.5	1000	9.3	99.6	L	[88]
Metal Oxide	GOF/Fe_3_O_4_	574.2	2.5	2	200	258.6	99.97	F	[143]
	G−ZnO	-	100	6	10	46.3	90	L	[90]
	NPG/Fe_3_O_4_	850	0.9	3	1000	43.5	80	F	[144]
	HR-M-GO/Fe_3_O_4_	182	1.6	7	300	31.8	100	L	[145]
	MGC	97	0.03	6.6	60	5.5	-	L	[146]
Metal Oxide + Organic	f-Fe_3_O_4_/G	60	0.07	3–4	600	280.6	-	L	[147]
	GCF	74.4	10	2	50	270.3	96	L	[72]
	CoFe_2_O_4_ -TETA-GO	-	4.4	2	100	180.1	-	L	[148]
	MCGO-IL	-	5	3	200	145.4	-	L	[149]
Organic	DAP–RGO	46.7	2	1	500	393.7	96	F	[150]
	Chitosan/GO	-	0.5	6	1000	310.4	>90	R–P	[38]
	Cs/CDTA-GO	-	-	3.5	25	167.0	90	L	[31]
	GEC	-	8	2	50	86.2	92.5	F	[141]
	GOSB	-	0.03	3	100	76.9	-	L	[151]
	GAD	37.6	0.001	4	200	72.7	-	L	[152]
	IT-PRGO	-	4	5	250	63	96	L	[47]
	MGNC	42.1	3000	2	1	1.0	100	-	[153]
Polymer	G-PDAP	327.8	4	1	500	609.8	100	L	[37]
	PmPD/rGO/NFO	-	0.4	3	-	502.5	-	L	[154]
	GO-PEI	1.2	-	-	-	436.2	-	L	[155]
	P(TA-TEPA)-PAM-RGO	-	-	6	-	387.5	-	L	[156]
	Fe_3_O_4_-GS	62.4	2.6	7.5	3	17.3	95	F	[77]
	Fe_3_O_4_/SiO_2_-GO-PEI	-	1	6	4	0.3	-	L	[140]

L: Langmuir, F: Freundlich, R–P: Redlich–Peterson.

All these high capacities have the initial adsorbate concentration greater than 200 mg/L. The ability to regenerate and reuse the GO-based adsorbents has been reported. In a few cases, Fe_3_O_4_/SiO2-GO-PEI was recycled several times without a significant decrease in its adsorption capacity [140]. GONF was reusable effectively for 4 cycles for the adsorption of Cr (III) [88] and rGO-NiFerrite NC could be easily reused and recycled for 4 cycles [30]. The most cycles of regeneration done was reported on GCF to achieve 90% of the capacity after 10 iterations. This shows the superior stability of GCF over the other adsorbents [72,149].

GEC’s adsorption capacity decreased slightly from 92.5% to 87.5% after 7 cycles, indicating it is suitable adsorbent for Cr (VI) removal [141]. For chitosan/GO, more than 90% of the total adsorption uptake in the first cycle is retained after the 5^th^ cycle of Cr (VI) removal [38]. Moreover, MCGO-IL retained 78% of the initial adsorption capacity after 5 cycles of Cr (VI) [148]. DAP–RGO was also recycled for five cycles and the maximum removal efficiency was reduced from 96% in the first cycle to 84% after the 5th cycle, indicating the good reusability [150]. The regeneration studies for PG-C and CNF-C revealed that the adsorbents had the potential to be reused for multiple cycles in the adsorption of Cr (VI) [85]. Upon analysis of regeneration studies and maximum capacities achieved, G-PDAP is recommended as a cost-effective adsorbent for the removal of chromium from aqueous solutions [37]. 

### 4.7. Copper Removal

Removal of copper ions or copper complexes from water are most widely studied among all other metal ions due to its extensive occurrence especially in the microelectronics industry effluents. Table 8 summarizes the results for the adsorption of Cu (II) onto GO and GO composites. Similar to the case of other metal ions, the isotherm results of Cu removal using graphene materials are mostly well described by the Langmuir isotherm, but with several exceptions where Tempkin or Freundlich isotherms provide the best fit for the equilibrium data. The results that follow the multilayer heterogeneous adsorption isotherms may be attributed to the tendency of the copper ions to adsorb by complexation with oxygen lone pair electrons on different oxygenated functional groups present on the surfaces of the adsorbents. On the other hand, the kinetic data are well described by the pseudo-2^nd^ order model. Only one study followed the Double Exponential kinetic model [38].

The highest copper uptake capacities shown in Table 7 are for A-Mgo (301 mg/g), β-CD/GPTMS/GO 352.7 mg/g, FA-mGO 283.29 mg/g, EDTA-MCS/GO (207.26 mg/g) [46], Chitosan/GO (423.8 mg/g), Cu(tpa).GO (235 mg/g), graphene oxide encapsulated polyvinyl alcohol/sodium alginate hydrogel microspheres (247.61 mg/g) <80%, GCAM10 (457.5 mg/g) and mGO (353.59 mg/g). Other graphene-based materials used for the removal of copper ions showed lower capacity including crumpled graphene balls (22.56 mg/g), Magnetic dithiocarbamate/rGO (113.64 mg/g), β-Cyclodextrin conjugated graphene oxide (117.07 mg/g), and dialdehyde cellulose grafted graphene oxide composite (65.1 mg/g).

**Figure 14:**
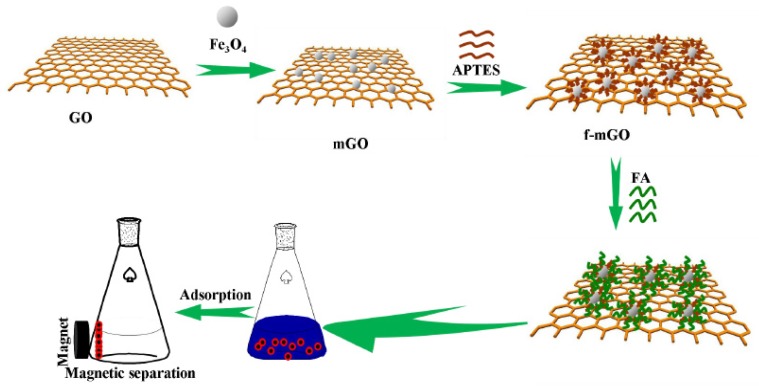
Synthesis, adsorption, and separation process of f-mGO (Reproduced or adapted from ref. [134], with permission from Elsevier, 2020).

In addition to high adsorption capacity, it is important for the expensive GO derived adsorbents to have the ability to be regenerated and reused many times, whilst retaining a high capacity. In this regard EDTA-mGO, FA-mGO, EDTA-MCS/GO, DTPA/MGO, GO-DPA, chitosan/GO, GO-CTPy, Fe_3_O_4_, Fe_3_O_4_/TiO2, CS-IIP and Fe_3_O_4_/HAP/GQDs were found to be reusable. EDTA-mGO retained a 97.6% removal efficiency after five cycles [83] and FA-mGO held 86.5% removal efficiency after six cycles, indicating that it is an efficient and economic adsorbent that can be easily retrieved and recycled due to it magnetic properties as shown in Figure 14 [134]. 

Magnetic graphene oxide is prepared through the Impregnation Method (mGO_i_), simple emulsion and Co-precipitation (mGO_p_) [21,50,61,71,134]. After synthesis, the adsorbent is characterized using various methods (SEM, FTIR, XRD, DTA, DTG, VSM) with results that show many possible interactions with the surface of this composite and its ability to remove copper [71]. 

Various methods were used to fabricate or synthesize the graphene composites used for the removal of copper. The three most common were solvothermal, hydrothermal and dispersion methods. In some cases, a combination of the methods was used to synthesize different components of the adsorbents [68,70]. The most common additive to graphene was organic, showing the most promise with regards to maximum capacities. Amino-MGO [118] reported the highest maximum capacity, although upon further analysis Chitosan/GO [38] is the recommended graphene composite for the removal of copper. It showed the best overall performance with regards to removal efficiency, regeneration properties, and adsorbent to adsorbate ratio. Furthermore, given that chitosan and graphene are abundant and not expensive compared to the other composites, it is the economical choice too. 

EDTA-MCS/GO showed high magnetic sensitivity in the presence of an external magnetic field after 4 cycles, which indicated good reusability status [46]. DTPA/MGO’s total adsorption capacity for Cu (II) decreased by only 8.2% after 6 cycles [39]. The removal efficiency of GO-DPA reduced with the number of cycles but was still above 80% after 3 cycles, indicating reasonably good reusability [40]. More than 90% of the total adsorption in the first cycle takes place in the 5^th^ cycle for metal ions [38]. The adsorption capacity of GO-CTPy was still high enough after seven cycles to indicate that it has good reusability performance [157]. It also had the best regeneration properties of all studied graphene composites for copper removal. The decline of adsorption capacities in Fe_3_O_4_ and FTG were 9% and 7.4% after 5 cycles [50]. CS-IIP’s adsorption capacity decreased slightly after 5 cycles, showing that CS-IIP has good stability and could be used repeatedly [86]. 

The adsorption capacities for copper using the more conventional adsorbents are generally much lower than the high values reported in Table 7. For example the adsorption capacity for chitosan (85 mg/g) [73], DTPA-chitosan/alginate composite beads (107 mg/g) [32], iminodiacetate ion exchanger (145 mg/g) [158], mixed adsorbents (238 mg/g) [159], poly-dopamine coated natural zeolite (28 mg/g) all with lower capacities than the 300+ mg/g capacity of the majority of graphene-based adsorbents shown in Table 7. The exception is the “superadsorbent” PAMAM CNT-dendrimers with a super-capacity of 3333 mg/g [160].

**Table 8:** Adsorption properties of different GO-based adsorbents used in Cu (II) removal.

Modification	Adsorbent	Surface Area (m^2^/g)	G/Cu Ratio (g/g)	pH	Max Cu Conc. (mg/L)	Capacity (mg/g)	Max. Removal (%)	Adsorption Isotherm	Ref.
Inorganic	GO –COOH	39.8	0.159	6–7	700	357.1	99.4	L	[161]
	BY-GO	34.2	0.674	5	493.3	349.5	-	L	[162]
	Cu(tpa).GO	-	0.04	7	228.1	235	-	L	[86]
	Graphene balls	-	-	3.5	249.7	224.6	99	F	[163]
	ZnO NR-rGO	-	5	8	98.6	67.4	100	L	[164]
Metal Oxide	GO-MO	383.9	0.02	6	500	240	99	F	[117]
	FTG	422.8	-	-	-	87.4	72.1	L	[165]
	SMGO	92.8	8.35	4.7	73.7	62.7	-	L	[166]
	G−ZnO	-	100	6	10	37.5	96.56	L	[90]
Organic	GCAM10	-	0.4	4.9	500	457.5	>85	F	[167]
	Chitosan/GO	-	0.5	6	1000	423.8	>90	R–P	[38]
	GO-DPA	-	0.4	5	100	358.8	>85	L	[40]
	β-CD/GPTMS/GO	-	1.25	7	200	352.7	-	L	[168]
	MWCNT-PDA	356.1	0.167	6	400	318.5	-	L	[91]
	SA/PVA/GO	-	-	8	-	247.7	>80	L	[159]
	EDTA-MCS/GO	49.9	6.6	7	50	207.3	86.8	L	[46]
	CS/GO_10_	-	0.1	5	100	202.5	-	F	[32]
	GAD	37.6	0.01	4	200	169.5	-	L	[152]
	GO-CTPy	57.3	16.7	6	90	119.6	-	L	[157]
	GOSB	-	0.3	3	100	111.11	-	L	[151]
	GO-TETA-DAC	445	-	5	-	65.1	77	L	[67]
	IT-PRGO	-	4	5	150	37	100	L	[47]
Organic + Metal Oxide	amino-MGO	-	-	5.5	-	578.1	-	L	[118]
	EDTA-mGO	49.9	1	4.1	100	301.2	95.7	T	[76]
	FA-mGO	-	2	5	2000	283.3	96.8	T	[134]
	Fe3O4@GO	169.7	0.05	5	1000	32.5	-	F	[169]
O+P	rGO-PDTC/Fe_3_O_4_	194.8	0.33	5	-	113.6	-	L	[68]
	g-C-EN-GO		0.26	7	959.2	46.4	43.1	L	[96]
Polymer	mGO/PAMAM2.0	-	-	7	30	353.6	99.9	F	[70]
	DTPA/MGO	-	0.033	3	300	131.4	-	L	[39]
	EDA-RGO	28	0.5	7	200	55.3	-	L	[99]
	MGO/β-CD	-	1.4	5.5	100	30.98	-	F	[170]
P + MO	GO/ Fe_3_O_4_/PEI	323.5	1	5	1000	157	97.1	L	[171]
	MCGON	132.9	0.167	7	100	217.4	-	L	[172]
	CS/GO/Fe_3_O_4_-IIP	-	0.6	6	500	132	-	F	[46]

L: Langmuir, F: Freundlich, R–P: Redlich–Peterson, T: Temkin, MO: Metal Oxide, P: Polymer, O: Organic.

### 4.8. Other Metals

Table 9 summarized the adsorption capacity, kinetics and experimental conditions for the adsorption of several important and toxic metals using GO and GO-based composites. All studies followed the pseudo second order kinetic model except PAA-MGA in the removal of selenium, which followed Intra-Particle Diffusion model [81]. These metals include radioactive materials. U, Th, Sc, Nd; catalytic materials such as Pd, Pt; emerging materials in waste streams including Se and Mn. For the adsorption of U (VI), GO-BSA showed good regeneration efficiency showing the potential for reusability [173]. Additionally, rGO/LDH’s adsorption capacity remained at 97.2% after three cycles [174]. While GO has high reusability, as its desorption percentages reached 95.45% for Au(III), 94.64% for Pd (II) and 99.37% for Pt(IV) [175]. MGO’s adsorption values can be compared to those for adsorbents that have been reported before, suggesting that it could be reusable [176]. The desorption percentage of CSGO_5_ remained about 95% with both Au(III) and Pd(II) after 3 cycles, showing that it is possible to successfully reuse CSGO_5_ for the adsorption of both metals [177].

## 5. Conclusions

GO provides an economically viable and efficient method to treat wastewater containing harmful heavy metals. Various methods are available for synthesizing composite GO materials providing materials that are tailored for removal of specific species. The carboxyl, hydroxyl, and epoxy functional oxygen groups on GO facilitate the adsorption of ions by providing active binding sites. This review analyzes the metal adsorption results from over one hundred and eighty recent studies using GO and GO-derived materials for the removal of more than 10 metal ions. The adsorption capacities of different metals are analyzed and compared with the capacity of conventional adsorbents. In almost every case, the metal adsorption capacity using GO materials are considerably higher. Although the production of GOs is expensive at present, these high capacities may lead to lower cost for treatment of wastewater streams polluted with metal ions as not only is the required adsorbent amount lower, but also the size of adsorption equipment will be less and hence the plant capital cost will be less. There are no studies that have considered how to recover the valuable metals for re-use which is an important topic that needs further research.

New research directions started in the last few years for GO hybridizations with different organics to enhance the metal uptake and speed the adsorption kinetics. More detailed mechanistic studies have been utilizing UV and ultrasound to facilitate the adsorption process. 

More research is required on new functional groups and composites that increase the maximum adsorption capacity of GO and improve its reusability. More fixed bed column studies are required and both testing of the graphene materials for selective removal of metals is still absent from the current literature as the current studies focus on testing single metals. Moreover, the effect of water salinity on the adsorption of heavy metals is important for treatment of oil- and gas-produced water, where high concentrations of monovalent salts exist. Another key issue is the ability to regenerate and retain the adsorption capacity of the GO-based adsorbent in an economic manner and recover the desorbed metal (in most cases as a precipitated pure salt) for reuse. There are almost no studies which have utilized the use of fixed bed columns experiments as almost all studies have been on batch experiments. Further studies in this area and scale-up are also required with estimation of the cost of the material and its impact on the economic viability of large-scale adsorption processes. 

## Figures and Tables

**Table 1:** WHO guideline for metal concentration in drinking water quality.

Metal	Cu	Cr	Cd	Hg	Pb	As	Ni
Permissible limit, µg/L	2000	50	3	3	10	10	700

**Table 2:** Adsorption properties of GO-based adsorbents used for removal of As (III) and As (V).

Modification	Adsorbent	Surface Area (m^2^/g)	G/As Ratio (g/g)	pH	Max. As Conc. (mg/L)	Capacity (mg/g)	Max Removal (%)	Ads. Isotherm	Ref.
-	GO	-	16.6	7.6	20	74.1	100	L	[44]
Organic	Epoxy-GNP NC	47.6	0.4	-	1000	190	<99	-	[45]
	Aminopyrazole-f-GO	-	16.6	7.6	20	131.6	100	L	[44]
	EDTA-MChitosn/GO	450	66	7	5	42.8	80.4	F	[46]
	Imino-Thiobiuret-rGO	-	4	5	100	19	100	L	[47]
Metal Oxide	G-Spinel CuFe_2_O_4_	-	2.4	4	1000	172.3	98	F	[48]
	FeO_x_-GO NC	341	0.7	7	1200	147	99.9	L	[49]
	Fe_3_O_4_/RGO/Cu- Zeolite	61.6	-	6	2000	50.5	-	-	[23]
	Fe_3_O_4_/rGO	109	2	5.5	100	54.5	100	L	[50]
	Magnetite-nonOxidG	190	100	7.1	1	38	-	S	[51]
Inorganic	Ag-Cu_2_O/rGO	-	1000	7	2	11.5	83.9	L	[50]
	LD-Hydroxide/GO	35.4	-	5	-	183.1	-	L	[52]
	GO-Mesoporous SiO_2_	873	1	-	100	47.3	97.7	L	[53]

L: Langmuir, F: Freundlich, S: Sips.

**Table 9:** Adsorption properties of different GO-based adsorbents used for removal of various metals.

Metal	Adsorbent	Surface Area (m^2^/g)	Adsorbent/Adsorbate (g/g)	pH	Max. Adsorbate Conc. (mg/L)	Capacity (mg/g)	Max. Removal (%)	Ads. Isotherm	Ref.
Ag (III)	TEOA-GO-PC	-	0.02	-	1000	850	-	L	[175]
Ag (III)	CNTs/GO	-	0.167	5.9	-	534.8	86.42	L	[77]
Au (III)	CSGO_5_	4.2	0.4	4	500	1077	-	L	[133]
Au (III)	GO	-	0.033	6	90	108.3	-	L	[133]
Ca (II)	pAMPS-g-GT	-	0.3	7	1000	114.2	53.45	L	[178]
Co (II)	GO	2.8	0.01	5.5	1000	21.3	93	F	[159]
Co (II)	M–GO	-	40	6.5	50	17.1	100	L	[176]
Co (II)	zero-valent FeNP-G	-	0.67	5.7	600	134.3	90	F	[109]
Co (II)	GO	-	10	5	10	43.6	-	S	[179]
Co (II)	ZnO NR-rGO	-	5	8	90.1	36.4	10	L	[164]
Co (II)	TRG	10	2	6	700	733	90	L	[81]
Fe (II)	MGO	-	0.36	5.5	84	43.2	100	F	[176]
Fe (III)	Cu(tpa).GO	-	0.14	7	73.7	78	-	L	[86]
Fe (III)	GO-PAMAM 2.0	-	-	-	-	29.7	-	-	[39]
Fe (III)	GO	330.7	0.6	-	20	133.3	90.5	L	[180]
Fe (III)	GO	-	-	4	25	27.3	-	L	[71]
Gd (III)	CNT/GO	-	4	5.9	12	427.7	-	L	[181]
Mn (II)	Cu(tpa).GO	-	0.09	7	112.4	150	-	L	[86]
Mn (II)	MGO	-	0.364	5.5	82.5	16.5	100	F	[176]
Mn (II)	EDA-RGO	28	0.5	7	200	42.5	-	L	[99]
Nd (III)	GO-C_4_	372	-	7	-	232.6	-	F	[135]
Nd (III)	GO-C_6_	518	-	7	-	220	-	F	[135]
Nd (III)	GO-C_8_	62.4	-	7	-	312	-	F	[135]
Pd (II)	CSGO_5_	4.2	2	3	100	216.9	-	L	[133]
Pd (II)	GO	-	0.06	6	50	80.8	-	L	[133]
Pt (IV)	GO	-	0.06	6	50	71.4	-	L	[133]
Re (VII)	ZrO_2_@rGO	272.73	-	4	-	43.6	-	L	[88]
Se (IV)	PAA-MGO	-	0.38	5.8	4	120.1	99.3	F	[42]
Se (IV)	PAA-MGO	-	-	3.1	0.4	156	99.3	F	[81]
Se (VI)	PAA-MGO	-	0.38	5.8	4	83.7	99.7	F	[159]
Sr (II)	GO–Hap ZEA	91.9	10	7	300	702.2	95	L	[29]
Th (IV)	a-GOM2	2001	0.25	3.8	1000	408.8	-	L	[98]
U (VI)	GO-BSA	-	-	6	200	389	98	L	[72]
U (VI)	a-GOM2	2001	0.5	3.8	1000	66.8	-	L	[98]
U (VI)	PA–GO	-	6.25	5.5	80	124.3	-	L	[182]
U (VI)	rGO/LDH	256.8	3.84	4	130	277.8	99	L	[72]
Zn(II)	GO	-	1.67	5	40	246	99.3	L	[183]
Zn (II)	GO	-	-	6	2439.1	-	94	L	[184]
Zn (II)	GOSO_x_R@TiO_2_	208	-	-	-	285	-	R-L	[83]
Zn (II)	GO	-	14.9	6.2	0.5	243	93.1	L	[185]
Zn (II)	Cu(tpa).GO	-	0.11	7	91.7	89	-	F	[86]
Zn (II)	GO-PANI	-	0.5	7	100	-	84.8	L	[184]
Zn (II)	GO-PAMAM 2.0	-	-	-	-	64.6	-	-	[39]

L: Langmuir, F: Freundlich, R–P: Redlich–Peterson, S: Sips.
